# Increased Prevalence of Breast and All-cause Cancer in Female Orthopaedic Surgeons

**DOI:** 10.5435/JAAOSGlobal-D-22-00031

**Published:** 2022-05-19

**Authors:** Loretta B. Chou, Brianna Johnson, Lauren M. Shapiro, Stephanie Pun, Lisa K. Cannada, Antonia F. Chen, Lindsey C. Valone, Sara S. Van Nortwick, Amy L. Ladd, Andrea K. Finlay

**Affiliations:** From the Department of Orthopaedic Surgery, Stanford University, Stanford, CA (Dr. Chou, Dr. Pun, Dr. Ladd); Walter Reed National Military Medical Center, Bethesda, MD (Dr. Johnson); the Department of Orthopaedic Surgery, University of California – San Francisco San Francisco, CA (Dr. Shapiro); Novant Health Orthopaedic Fracture Clinic, Charlotte, NC (Dr. Cannada); the Department of Orthopaedic Surgery, Harvard Medical School Boston, MA (Dr. Chen); the Department of Orthopaedic Surgery, California Pacific Orthopaedics San Fransciso, CA (Dr. Valone); the Department of Orthopaedic Surgery, Medical University of South Carolina Charleston, SC (Dr. Van Nortwick); and the Department of Orthopaedic Surgery, Palo Alto Veterens Association Palo Alto, CA (Dr. Finlay).

## Abstract

Cancer is the second leading cause of death among women in the United States. Previous studies demonstrate a higher prevalence of cancer among female orthopaedic surgeons. This study aimed to provide an updated prevalence of breast and all-cause cancer among female orthopaedic surgeons using a larger and more current study population.

**Methods:**

We distributed surveys to female orthopaedic surgeons in national orthopaedic specialty societies. Six hundred seventy-two survey responses were collected. We calculated standardized prevalence ratios (SPRs) and 95% confidence intervals (CIs) based on gender-specific, race-specific, and age-specific cancer prevalence statistics in the US population. We compared the distribution of breast cancer risk factors with that of women in the 2018 and 2009 California Health Interview Survey.

**Results:**

Fifty-one of the 672 surveyed surgeons reported a diagnosis of invasive cancer. Twenty reported breast cancer with a prevalence higher among female orthopaedic surgeons compared with the US female population (SPR: 2.89, 95% CI: 2.16 to 3.81, *P* < 0.001). The breast cancer prevalence was also higher among orthopaedic surgeons compared with the US female population (SPR: 3.97, 95% CI: 2.43 to 6.14, *P* = 0.003).

**Discussion:**

The increased prevalence of breast and all-cause cancer among a larger and more diverse cohort of female orthopaedic surgeons confirms previous studies and provides an update regarding a concerning public health issue within this specialty.

Cancer is the second leading cause of death among women in the United States (US), accounting for 20.7% of deaths in women. Breast cancer is the second leading cause of cancer-related deaths among women.^1,2^ According to data from the Surveillance, Epidemiology, and End Results Program (SEER), the National Program of Cancer Registries, and the North American Association of Central Cancer Registries, almost 13% of women will be diagnosed with breast cancer at some point during their lifetime, and approximately 1.1% of women were living with breast cancer in the United States in 2017.^1,3^ Thus, improvements in breast cancer prevention have the potential to profoundly affect women's health.

Previous studies have shown a higher prevalence of breast cancer in female orthopaedic surgeons compared with the general US female population.^4,5^ Among female orthopaedic, urology, and plastic surgeons, orthopaedic surgeons are the only subspecialty to demonstrate a higher prevalence of breast cancer than expected.^6^ These previous studies have shown an 85% higher prevalence in all-cause cancer and a 190% higher prevalence in breast cancer among female orthopaedic surgeons compared with the general female population.^4-6^

Previous studies reporting a higher prevalence of breast cancer among orthopaedic surgeons were limited by sample breadth and size and were conducted more than 10 years ago. Thus, this study aimed to report an updated prevalence of all-cause cancer and breast cancer among female orthopaedic surgeons by using a more current, larger, and broader study population. We hypothesized that female orthopaedic surgeons would have a higher prevalence of breast and all-cause cancer than the general US female population.

## Methods

### Survey Methods

Between February 2019 and February 2020, we contacted 1290 female orthopaedic surgeons through e-mail or the websites of national orthopaedic specialty societies and asked them to complete a survey. Of these surgeons, 672 participated in the study (52% response rate). The surveys were sent to the following orthopaedic specialty societies: the Ruth Jackson Orthopaedic Society (RJOS), Pediatric Orthopaedic Society of North America, Musculoskeletal Tumor Society, Orthopaedic Trauma Association, American Association of Hip and Knee Surgeons Women in Arthroplasty Committee, American Orthopaedic Foot and Ankle Society, American Orthopaedic Society for Sports Medicine, and American Society for Surgery of the Hand.

The survey was based on previous studies and follows the principles for conducting surveys with orthopaedic surgeons described by Sprauge et al.^4,5,7^ The survey was initially developed with a cancer epidemiologist in 2010. It was used in previous studies and subsequently revised and pilot-tested by multiple experts for this study.^4,5^ We provided a cover letter explaining the purpose of the survey, as well as a link to the survey. The survey was created in Research Electronic Data Capture, a secure web application used for building and managing surveys online. We used this encrypted survey through qualtrics. The survey participants were contacted through subspecialty societies and RJOS. To increase response rates, surveys were sent again to some society members, following each society's protocol. These societies included RJOS and Arthroplasty Committee, American Orthopaedic Foot and Ankle Society.

Demographic survey questions collected information regarding age, race/ethnicity, body mass index, smoking history, menstrual and reproductive history, and use of hormones. Professional history questions focused on subspecialty, years in practice, use of fluoroscopy, polymethylmethacrylate (PMMA) use, lead protection, dosimeter use, and training or education on radiation exposure risk. Cancer diagnoses were self-reported based on the following questions: Have you ever been diagnosed with breast cancer? Have you ever been diagnosed with cancer other than basal or squamous cell carcinoma of the skin? What type of cancer were you diagnosed with? When were you first diagnosed with cancer? We also obtained a family history of cancer. This study was approved by the Stanford Institutional Review Board.

### Statistical Analysis

We obtained descriptive statistics regarding the clinical practice characteristics of female orthopaedic surgeons, including employment history, setting, subspecialty, and occupational radiation exposure, which was estimated by standard fluoroscopy use, minifluoroscopy use, and PMMA use. Breast cancer risk factors were compared between female orthopaedic surgeons and women in the 2018 and 2009 California Health Interview Survey (CHIS). The CHIS is a population-based survey conducted through telephone and is representative of the noninstitutionalized population in households.^8^ Adult CHIS surveys include questions on demographics and women's health, such as age at first childbirth, number of births, and hormone replacement therapy. Samples from the 2018 and 2009 CHIS were limited to women aged 30 years or older to ensure similarity to the female orthopaedic surgeon data. We used a report on the US female population to obtain the total fertility rate for 2018.^9^ Both 2018 and 2009 CHIS samples were included because different questions that represented risk factors for breast cancer were asked each year.

Cancer prevalence analyses were limited to invasive internal cancers and melanomas diagnosed in female orthopaedic surgeons within 17 years of survey completion date. We selected the 17-year cutoff to align cancer rates among orthopaedic surgeons with the 17-year prevalence statistics (2000 to 2017) collected by SEER.^10^ SEER prevalence statistics represent existing and new cancer cases for people who were alive on a certain date. We excluded four cases of cancer (two cases of lymphoma and two cases of thyroid cancer) that were diagnosed more than17 years ago and excluded two diagnoses of basal and squamous cell cancer because these are excluded from SEER. We examined both the total samples of female orthopaedic surgeons and only currently practicing surgeons. Based on their potential for occupational radiation exposure, we categorized the surgeons into two groups: high and low exposure. Surgeons who more frequently imaged thick, bony, or muscular areas (general orthopaedics, oncology, shoulder, spine, and trauma) were considered to have a greater opportunity for radiation exposure and were categorized as high exposure. We compared cancer prevalence in the high-exposure group with that for all orthopaedic subspecialties with potentially lower radiation exposure. We compared cancer prevalence among surgeons by subspecialty, fluoroscopy use frequency, and protective shield use. To estimate the lifetime breast cancer risk for a representative woman in our study population, we used the National Cancer Institute Breast Cancer Assessment Tool (i.e., the Gail model).^11^

We calculated standardized prevalence ratios (SPRs) by dividing the observed number of cancer cases among female orthopaedic surgeons by the expected number derived from age-specific, gender-specific, and race-specific cancer prevalences observed in the general US female population. To calculate confidence intervals (CIs) and *P*-values, we assumed a Poisson distribution for the observed number of cases and used an approximation to the exact Poisson test, as performed in previous studies.^4,5,12^

We conducted sensitivity analyses to examine the effect of potential response bias in our results, assuming that women without a cancer diagnosis may be less likely to participate. We calculated SPRs and 95% CIs based on the following assumptions: (1) no nonrespondents had a history of cancer and (2) nonrespondents had the same cancer prevalence as the US female population. Analyses were conducted in R version 3.6.3 and Rstudio.^13,14^

## Results

Of the 672 female orthopaedic surgeons who completed the survey, 87.5% reported working at least part-time and the mean duration of employment was 16 years (Appendix Table A1, http://links.lww.com/JG9/A218). More than half of the surgeons (55.8%) used standard fluoroscopy more than once a week. Minifluoroscopy use was less common, with 34.5% of respondents using it fewer than six times per year and 29.9% using it more than once a week.

We compared breast cancer risk factors of female orthopaedic surgeons with those of population-based samples of women in California and the United States (Table 1 and Appendix Table A2, http://links.lww.com/JG9/A219). Compared with the general California female population, surgeons in our sample were more likely to be younger, have a normal body mass index (<25.0 kg/m^2^), be White, and have never smoked, which are deemed protective factors for breast cancer.^5,15-17^ Regarding hormone-related breast cancer risk factors, fewer surgeons had used hormone replacement therapy, and the surgeon respondents took hormones for a shorter duration than the California female population. Age at first childbirth was older for surgeons than the comparison group, which is considered an increased risk for breast cancer.^15-17^

**Table 1:** Distribution of Breast Cancer Risk Factors Among US Female Surgeons

Characteristic	Orthopaedics (n = 672)	CHIS Women or US Population
Mean age (SD), yr^a^	42.71 (9.76)	59.07 (14.98)
Median age (IQR), yr^a^	39.93 (12.96)	59.00 (23.00)
Age range, yr^a^	26-72	30-85
Age categories, n (%)^b^		
<40	321 (47.8%)	987 (9.7%)
40-49	129 (19.2%)	1227 (12.0%)
50-59	29 (4.3%)	1866 (18.3%)
≥60	163 (24.3%)	9138 (60.1%)
Missing	30 (4.5%)	
Race/ethnicity, n (%)^b^		
White	524 (78.0%)	6489 (63.5%)
Asian/Asian Pacific Islander	47 (7.0%)	807 (7.9%)
African American	18 (2.7%)	581 (5.7%)
Ashkenazi Jewish	8 (1.2%)	—
Hispanic	11 (1.6%)	1830 (17.9%)
Other or mixed	50 (7.4%)	511 (5.0%)
Missing	14 (2.1%)	—

a 2009 California Health Interview Survey (n = 26,032).

b California Health Interview Survey 2018 sample (n = 10,218).

Fifty-one women reported a diagnosis of invasive cancer, and 20 reported a diagnosis of breast cancer (Table 2). Cancer prevalence was lower (2.6%) among surgeons with a subspecialty that was likely to have higher radiation exposure compared with surgeons in other specialties (8.7%, *P* = 0.016), but the prevalence did not differ by individual subspecialty. Cancer prevalence did not differ by frequency of use of fluoroscopy, PMMA, or protective shielding (Table 3). Furthermore, we did not detect a relationship between all-cause cancer prevalence and standard or minifluoroscopy use. However, specifically for breast cancer, the prevalence rate was significantly different between groups categorized by the type of fluoroscopy most commonly used (*P* = 0.015). Surgeons who used both standard and minifluoroscopy had a 5.6% breast cancer prevalence rate compared with surgeons who primarily used minifluoroscopy (6.4%), standard fluoroscopy (1.1%), or neither (2.4%). Post hoc tests indicated significant differences between surgeons who use both compared with those using only standard fluoroscopy (*P* = 0.018) and surgeons who use minifluoroscopy compared with those using standard fluoroscopy (*P* = 0.011).

**Table 2:** Self-reported Cancers Among Female Orthopaedic Surgeons (n = 672)

	n (%)
Total cancer/carcinoma in situ (CIS)	51
Site of cancer	
Acute promyelocytic leukemia	1 (2.0%)
Breast	20 (39.2%)
Colon	4 (7.8%)
Endometrial	3 (5.9%)
Lymphoma	1 (2.0%)
Melanoma	10 (19.6%)
Multiple myeloma	4 (7.8%)
Thyroid	1 (2.0%)
Tongue cancer	1 (2.0%)
Did not specify	6 (11.8%)

Two participants reported both breast cancer and melanoma, both diagnoses are counted in the table.

**Table 3:** Distribution of Cancer Prevalence by Frequency of Fluoroscopy, Polymethylmethacrylate, and Protective Shielding Use Among Female Orthopaedic Surgeons (n = 672)

	Self-reported history of invasive cancer		*P*
	Yes (n = 35)	No (n = 602)	
Standard fluoroscopy use			0.590
Almost never or ≤ 4 times/mo	15 (42.9%)	227 (37.7%)	
>1 time/wk	19 (54.3%)	356 (59.1%)	
Minifluoroscopy use			0.266
Almost never or ≤4 times/mo	20 (57.1%)	396 (65.8%)	
>1 time/wk	14 (40.0%)	187 (31.1%)	
Fluoroscopy use with procedures lasting > 10 min	(n = 2)	(n = 34)	0.524
Almost never or ≤4 times/mo	1 (50.0%)	10 (41.7%)	
>1 time/wk	1 (50.0%)	24 (70.6%)	
Polymethylmethacrylate (PMMA) use			0.079
Almost never or ≤4 times/mo	29 (82.9%)	412 (68.4%)	
>1 time/mo	5 (14.3%)	169 (28.1%)	
Current protective shielding use			0.488
Almost never or ≤4 times/mo	3 (8.6%)	38 (6.3%)	
>1 time/wk	31 (88.6%)	544 (90.4%)	

N = 20 missing for fluoroscopy; n = 22 missing for PMMA; n = 21 missing for current lead protection.

The prevalence of cancer was 189% higher among female orthopaedic surgeons compared with that of the age-adjusted and race-adjusted general US female population (Appendix Table A3, http://links.lww.com/JG9/A220). The prevalence of breast cancer was fourfold higher among women in our sample compared with the age-adjusted and race-adjusted US population of women. When the sample is restricted to currently practicing orthopaedic surgeons, the prevalence was still markedly higher. Using the Gail model, we estimated the lifetime breast cancer risk of a hypothetical woman in our sample (e.g., no medical or family history of breast cancer, aged 43 years, and White race/ethnicity, aged 12 to 13 years at menarche). The risk was 10.8% for women in our sample compared with 12.1% for matched women in the general US population (CIs are not provided for the Gail model breast cancer risk assessment tool).

We calculated SPRs and 95% CIs with the assumption that respondents and nonrespondents were of the same race/ethnicity and age. The first set of sensitivity analyses assumed that none of the nonrespondents had a history of all-cause cancer or breast cancer. The second set of sensitivity analyses assumed that nonrespondents had the same prevalence of all-cause cancer and breast cancer as the general US female population. These analyses indicated that the overall cancer prevalence and breast cancer prevalence remained higher for female orthopaedic surgeons compared with the general US population of women. For overall cancer prevalence, the SPR was 1.51 (95% CI: 1.12 to 1.98) when we assumed that there were no cancer cases among nonrespondents and 1.99 (95% CI: 1.54 to 2.52) when we assumed that the prevalence of cancer cases among nonrespondents matched that of the general US female population. In sensitivity analyses for breast cancer prevalence, the SPR was 2.07 (95% CI: 1.26 to 3.20) when no cases were assumed for nonrespondents and 2.55 (95% CI: 1.64 to 3.77) when population-expected rates were assumed for nonrespondents.

## Discussion

With an increasing percentage of women in orthopaedic surgery, it is imperative that we study and understand the occupational risks for this population to mitigate risks.^18,19^ Our study reports a 1.89-fold higher prevalence of all-cause cancer and a 3.97-fold higher prevalence of breast cancer in female orthopaedic surgeons compared with matched US women. Furthermore, our results are in contrast to the Gail model, which indicates a lower risk for breast cancer in our sample than the general population, indicating our results are even more surprising than expected. The finding of a higher cancer prevalence in a larger and more diverse cohort of female orthopaedic surgeons, which drew from a larger pool of female orthopaedic surgeons and recruited from more surgical societies than previous studies. The findings confirm previous studies and emphasize the importance of cancer as a public health issue within this specialty.^4-6^

The many reasons proposed for this higher prevalence include higher socioeconomic status, delayed childbearing, lifestyle factors, and occupational exposures.^4,20,21,22,23,24,25,26,27,28^ In a systematic review and meta-analysis, Lundqvist et al.^24^ demonstrated a higher incidence of breast cancer in patients with higher socioeconomic status (relative risk: 1.25, 1.17 to 1.32). Although we did not collect data regarding socioeconomic status, surgeons are in the top 1% of US wage earners.^3^ Higher socioeconomic status has been correlated with both lower parity and nulliparity and greater age at first childbirth, and all factors associated with a greater risk of breast cancer and all observed in our population.^4,21,29,30^

Occupational exposure to ionizing radiation is common in orthopaedic surgery. Fluoroscopy times are generally higher for less experienced surgeons; thus, notable radiation exposure may occur during training and early practice.^31^ Surgeons in training may be less likely to have access to appropriately fitting personal lead protection garments due to the costs and logistics of covering multiple hospitals. In particular, female trainees may be less likely to fit hospital-owned lead protection garments supplied by hospitals. Additional study has demonstrated that the addition of lead sleeves and/or axillary supplements decrease intraoperative lead exposure to the breast tissue most commonly affected by cancer.^32^

In our study, the prevalence of cancer did not differ by individual subspecialty. Previous studies have demonstrated that those specializing in trauma are exposed to higher rates of radiation.^33,34^ In this study, increased fluoroscopy use was not associated with an increased breast cancer prevalence. We also did not detect an increased breast cancer risk by radiation exposure group or specialty type; however, the sample size may have been too low to detect differences.

Researchers have not yet clarified the effect of decades-long exposure to low-dose radiation on the risk of breast cancer. Occupational radiation exposure is a modifiable risk factor that we can immediately change in practice. We therefore advocate for protective lead shielding of all radiation-sensitive tissue with appropriately fitted radiation-protective garments. Valone et al.^28^ used an anthropomorphic model to evaluate factors associated with higher breast radiation exposure and found that incorrectly fitting lead aprons and positioning relative to the c-arm affect the exposure rate for the upper outer quadrant of the breast. In addition, additional evaluation studying breast radiation with the use of appropriately fitting lead vests demonstrates that the upper outer quadrant of the breast, which is the most common site of breast cancer, is not well protected in many simulated intraoperative scenarios.^28,32^

The female orthopaedic surgeons in our study had lifestyle factors protective against cancer. Compared with the general female population in California and the United States, more women in our study had a normal body mass index, were nonsmokers, and had lower exposure to hormone replacement therapy.^15-17^ Despite these protective factors, female orthopaedic surgeons still reported a higher prevalence of cancer compared with matched US women. Additional investigation is needed to further delineate these variables and their correlation with cancer risk.

This study has limitations. For instance, healthier surgeons are likely to continue working, whereas a surgeon who has cancer may discontinue working and cease membership in a professional society. Thus, our recruiting mechanism may have introduced selection bias. Another limitation is recall bias inherent in the use of self-reported information. Although not ideal, it is unlikely that the study participants inaccurately reported cancer diagnoses, given the high education of women who participated in this study. A woman with a history of cancer may be more aware of her radiation exposure and may more accurately report her fluoroscopy use and be more vigilant with protective shielding. We did not observe a consistently different frequency of use of fluoroscopy types among orthopaedic surgeons, despite a markedly higher cancer prevalence; this finding suggests that if recall bias did exist, it was minimal. Relatedly, we used categorical criteria to evaluate fluoroscopic use (almost never or ≤4 times/mo vs >1 time/wk) which limits analyses. Studies demonstrate differential use of fluoroscopy and radiation exposure^34^ which would not be captured in our analysis (e.g., trauma surgery vs oncologic surgery). In addition, there was no information on radiation exposure among the matched US cohort; therefore, we could not conduct comparisons between this group focused on radiation exposure. As another limitation, a roster of all female AAOS orthopaedic surgeon members was not available. We used specialty society rosters that may be incomplete. Compared with the 2018 AAOS Orthopaedic Practice in the US Census Report, which had a survey response rate of 22.5%, our study response rate was 52% and represents an estimated 39% of all female surgeons in the United States. Although the response rate was not as high as in our previous studies (52% vs 81.1%^4^ and 69.7%^5^), the results were similar across studies indicating that orthopaedic surgeons are still at higher risk of breast cancer. Also of note, although the use of fertility and assistive reproductive technologies are noted to be needed in more than 10% of the female orthopaedic surgeons,^35^ we did not inquire about fertility treatment nor did we evaluate the effect of fluoroscopy use on fertility treatment or the effect on various types of cancers. Finally, our study was limited to female orthopaedic surgeons, but male orthopaedic surgeons experience similar occupational hazards and may be at elevated risk for breast cancer.^36^ Studies including male surgeons are warranted.

Our findings strongly suggest that female orthopaedic surgeons are at a higher risk of breast cancer than the general US female population. A likely contributory factor may be occupational exposure to ionizing radiation. The potential adverse health effects among surgeons caused by a cumulative low dose of ionizing radiation from fluoroscopy use during surgery are unknown. In addition, the higher breast cancer prevalence may be partly due to delayed childbearing and lower parity. Although additional research is needed to determine the factors behind the increased cancer risk among orthopaedic surgeons, the community should educate current practitioners in the use of protective shielding. Such protective factors will help prevent all cancers, including breast cancer.
